# Coping strategies, resilience and quality of life: reaction to the COVID-19 pandemic among Romanian physicians

**DOI:** 10.1186/s12960-024-00909-w

**Published:** 2024-05-07

**Authors:** Cătălina Angela Crișan, Răzvan Pop, Roland Stretea, Zaki Milhem, Alina-Ioana Forray

**Affiliations:** 1 Department of Neurosciences, Discipline of Psychiatry and Pediatric Psychiatry, Iuliu Hațieganu, University of Medicine and Pharmacy, 400349 Cluj-Napoca, Romania; 2 Clinical Hospital for Infectious Diseases, 400347 Cluj-Napoca, Romania; 3 Department of Community Medicine, Discipline of Public Health and Management, Iuliu Hațieganu University of Medicine and Pharmacy, 400349 Cluj-Napoca, Romania

**Keywords:** Mental health, Physicians, COVID-19, Resilience, Coping behavior, Quality of life, Cross-sectional studies, Psychological stress, Health behavior

## Abstract

**Background:**

The COVID-19 pandemic has presented multiple psychological challenges for healthcare workers, such as anxiety, depression, burnout, and substance use disorders. In this research, we investigate the different ways Romanian physicians dealt with the difficult period of the COVID-19 pandemic. We also analyze how positive and negative stress-reducing strategies, as well as demographic variables, affect their psychological resilience and quality of life. Our goal is to provide a comprehensive overview of how physicians coped with the unprecedented global health challenges.

**Methods:**

We carried out a national cross-sectional study of 265 physicians in Romania between January 2021 and January 2022 using a web-based questionnaire. The study employed a web-based questionnaire to assess coping mechanisms using the COPE inventory, resilience through the Connor-Davidson Resilience Scale 25 (CD-RISC 25), and quality of life via the WHOQOL-BREF scale. The COPE inventory, consisting of 60 items across 15 subscales, categorizes coping strategies into problem-focused, emotion-focused, and dysfunctional types, with each item rated on a 4-point scale. The CD-RISC 25 measures resilience on a 5-point Likert scale, with total scores ranging from 0 to 100. WHOQOL-BREF assesses quality of life through 26 items in 4 domains: physical, mental, social relations, and environmental, scored from 1 to 5 and converted to a 0–100 scale for domain scores. Univariate and multivariate linear regression models were employed to discern the intricate relationships between coping strategies, resilience levels, quality of life dimensions, and pertinent demographic factors.

**Results:**

The average CD-RISC score among participants was 66.2. The mean scores for the values for the QOL subscales were 64.0 for physical well-being, 61.7 for psychological well-being, 61.2 for social relationships, and 64.7 for environment. Individuals tend to use problem-focused and emotion-focused coping more than dysfunctional mechanisms, according to the COPE inventory. Problem-focused and emotion-focused coping are positively correlated with resilience, while dysfunctional coping is negatively correlated. Resilience is significantly influenced by gender and professional status, with males and senior specialists reporting higher levels while younger physicians and residents reporting lower levels.

**Conclusions:**

Our data points to specific protective characteristics and some detrimental factors on physicians' resilience and quality of life during the pandemic.

## Background

According to data from the World Health Organization (WHO) reporting data, between 2019 and 2023, the coronavirus disease 2019 (COVID-19) affected more than 772 million people and claimed more than 6.9 million lives [1]. Because of the pandemic, the world has been experiencing an unprecedented health crisis and drastic changes to personal and professional lives due to worldwide panic, fear, and anxiety [2, 3]. Despite extensive protective measures and immunization, the SARS-CoV-2 virus is still responsible for more than 800 thousand deaths worldwide in 2023 [4]. The pressure on the healthcare system persists, leading to the overburdening of health services and medical staff, especially in emergency departments [5–7]. In the Romanian healthcare context, the COVID-19 pandemic has illuminated the vulnerabilities and challenges in the health system, particularly in emergency preparedness and service delivery. The study by Stafie et al. (2021) provides insights into the Romanian healthcare system's response, highlighting the increased workload and lack of protection and well-being among physicians [8, 9]. Research shows that frontline healthcare workers (HCWs) who are involved in direct patient care during a pandemic experience greater anxiety and have more serious consequences due to mental and physical exhaustion, longer working hours, and the threat of infecting their families [10]. Hence, HCWs are putting their physical and psychological integrity at risk to stop the spread of COVID-19. According to the literature, medical personnel with direct engagement in COVID-19 management and lack of adequate psychological support were more likely to develop personal, work, and patient-related burnout [11, 12]. The pandemic has raised multiple psychological challenges among HCWs. Such pressure may encourage the silent development of mental health issues such as depression, substance use, or post-traumatic stress disorder [13, 14]. The never-ending ethical dilemmas, loss of colleagues or loved ones, family problems due to overworking, and other professional and personal conflicts have drastically lowered the life quality of medical staff [15, 16]. In this context, the concept of resilience, particularly in healthcare workers during the COVID-19 pandemic, has been a focal point in recent research. Resilience refers to the capacity of individuals to manage hardships and flourish in their personal and social lives [17]. This dynamic process is influenced by individual traits and environmental factors [18]. Psychological resilience, in the context of a crisis, is the ability to cope emotionally and return to a pre-crisis state [19]. In the healthcare setting, resilience has been characterized as a multidimensional psychological trait that enables individuals to thrive in the face of adversity [20]. Several studies focused on the psychosocial impact of COVID-19 on healthcare workers, identifying resilience as an essential factor in adapting to and overcoming stress [21, 22]. Thus, HCWs' resilient conduct in a stressful workplace has been linked to a higher quality of life and better health [23]

The present study is based on the transactional stress/coping model. This model posits that individuals’ coping responses and psychosocial resources critically influence health and stress outcomes, emphasizing the need to study the personal psychological resources of workers to maintain health despite exposure to adverse conditions [24]. The transactional theory of stress is a useful framework to comprehend the impact of the pandemic on healthcare providers. According to this theory, individuals are not passive recipients of stress, but active agents who can deploy personal psychological resources to cope with adverse conditions. Therefore, it is important to study the psychological well-being of healthcare workers to maintain their health in the face of challenging circumstances [25].

A systematic review from 2021 underscores the significance of coping behaviors, resilience, and social support in HCWs' mental health during the COVID-19 pandemic. Coping strategies are divided into problem-focused, emotion-focused, and dysfunctional types. Increased coping strategies and building resilience are considered protective factors against the mental health consequences of a stressful situation. For instance, resilient individuals are more likely to engage in positive coping activities, which buffer the negative impact of work-related stress on mental health [26]. However, given the ongoing impact of the COVID-19 pandemic, there is a need to identify helpful psychosocial factors and resources that can mitigate the impact of stress during emergent crises. The quality of life (QOL) is influenced by an individual's subjective experiences and environmental interactions, being shaped by cultural and personal value systems [27]. Hobfoll's Conservation of Resources Theory further elucidates this by linking QOL to personal resource levels, suggesting that managing and augmenting resources like resilience and coping skills enhances life satisfaction and health, particularly in high-stress environments [28].

Previous findings suggest that demographic variables such as age, gender, educational qualifications, marital status, living residence, and professional background have been shown to influence resilience and the domains of quality of life among healthcare workers during the COVID-19 pandemic [29–31]. Therefore, the present study, aims to offer insights into the relationship between sociodemographic characteristics of Romanian physicians and their psychological resilience and quality of life.

After COVID-19 was declared a pandemic on 11th March 2020 by WHO, each government started establishing restrictions to minimize transmission of the SARS-CoV-2 virus. All measures have inevitably increased mental health pressure among HCWs, who now had both their professional and personal lives affected by the pandemic [32–34]. Romania's government followed WHO containment guidelines. However, many residents returning from overseas, insufficient healthcare system infrastructure, and sociocultural variables raised obstacles at various stages of the epidemic's containment [35, 36]. Because the pandemic stretched the Romanian healthcare system far beyond its limit, the need to identify proper coping and resilience mechanisms for HCWs is urgent [37, 38].

The primary objective of this research is to thoroughly investigate the factors that affected the resilience and quality of life (QOL) of Romanian physicians during the state of emergency due to the COVID-19 pandemic in 2021. We aim to elucidate how different coping strategies, as evaluated by the COPE inventory, helped in alleviating the psychological and work-related stresses experienced by the physicians who participated in our study. By employing a cross-sectional study design, we explored how demographic factors and coping strategies together are associated with resilience and the four QOL domains as defined by the WHO: physical, psychological, social, and environmental health. Moreover, the research analyzed the association between resilience and the QOL domains. Additionally, the study aimed to document the patterns of substance use and psychiatric morbidity among these physicians during the COVID-19 pandemic.

## Methods

### Study design and subject recruitment

Between January 2021 and January 2022, we conducted a cross-sectional study that aimed to assess the resilience of Romanian-speaking physicians who lived and worked in Romania, in coping with the COVID-19 pandemic and how it affected their quality of life. We used an anonymous survey of 124 questions distributed to physicians through e-mail lists and social media. The study participants were over 18 years old and were all physicians. Exclusion criteria included medical students and other types of healthcare workers.

### Questionnaires used in data collection

The first section of the questionnaire included questions related to sociodemographic characteristics. Participants were queried about various demographic attributes such as age, gender, educational qualifications, marital status, living residence, and professional background. The second section of the questionnaire referred to preexisting mental health disorders, substance use behaviours, and psychiatric examinations during the emergency state.

The third section of the questionnaire addressed participants' resilience, which was measured using the Romanian version of the 25-item Connor-Davidson Resilience Scale (CD-RISC-25). Developed by Connor and Davidson in 2003, the scale consists of 25 items, each rated on a 5-point Likert scale (0–4), with higher scores reflecting greater resilience. The total score can range from 0 to 100 [39].

The fourth questionnaire section included the World Health Organization Quality of Life Scale-Short form (WHOQOL-BREF). The scale consists of 26 questions, scoring between 1 and 5. It has four subscales: physical domain (containing 7 items), psychological domain (containing 6 items), social relations domain (containing 3 items), and environmental domain (containing 8 items). Each item is rated on a 5-point scale. The mean score of items within each domain is used to calculate the domain score. Mean scores are then multiplied by 4 in order to make domain scores comparable with the scores used in the WHOQOL-100 scale, and subsequently transformed to a 0–100 scale. The quality of life increases as the score gets higher. The last section evaluated data concerning the coping mechanisms of study participants with the Romanian version of the COPE inventory. It comprises fifteen 4-item scales grouped into three categories: problem-focused, emotion-focused, and dysfunctional coping. The COPE Inventory consists of 60 items and 15 subscales, which include acceptance, active coping, behavioral disengagement, denial, seeking emotional support, seeking instrumental support, mental disengagement/self-distraction, planning, positive reinterpretation, religion, restraint, substance use, suppression of competing activities, venting, and humor [40].

### Statistical analysis

In this cross-sectional analysis, demographic variables, substance use behaviors, and psychiatric morbidity were summarized using descriptive statistics with frequencies and percentages. Means, standard deviations, medians, and interquartile ranges were employed for continuous variables. All analyses were performed on SPSS Software (version 29; MacOS).

Initially, each independent variable was examined separately for its association with resilience (CD-RISC scores) and quality of life (WHOQOL-BREF domains). This step helped in understanding individual variable effects. Following the univariate analysis, we utilized the 'Enter' method for the multivariate linear regression analysis. This approach was chosen due to its ability to simultaneously consider all identified predictor variables, each potentially influential on our outcomes of interest: resilience (CD-RISC scores) and quality of life (WHOQOL-BREF domains). The 'Enter' method is particularly effective in exploratory studies where predictors are identified individually from the literature, allowing us to assess their collective impact in a single model. Unstandardized coefficients (B) with 95% confidence intervals (CI) were reported for both univariate and multivariate analyses. To ensure the robustness of the multivariate regression analysis, we checked for multicollinearity among independent variables. Statistical significance was set at p-values of less than 0.05 for *, less than 0.01 for **, and less than 0.001 for ***. Independent variables for resilience included coping strategies categorized as problem-focused, emotion-focused, and dysfunctional coping, demographic variables (sex, age, marital status, education level, and area of residence), and professional status. In the case of the physical domain, mental domain, social relations domain, and environmental domain of QOL, CD-RISC was additionally added as an independent variable.

## Results

A total of 367 individuals completed the questionnaire, but 102 were excluded (27.79%) as they identified as other health professionals or as students in health sciences programs. The demographic data of the survey participants is presented in Table 1. Out of the respondents, 265 physicians participated in the survey. The study population predominantly comprises females (84.5%) between the ages of 31 and 50 years (54.7%), mostly residing in urban areas (92.1%). A slight majority hold advanced academic qualifications (55.1%), and the participants' professional backgrounds are diverse, with senior specialist physicians being the most prevalent group (37.4%). Most participants have been married at some point (63.8%).

**Table 1:** Socio-demographic and professional characteristics of study participants (n = 265)

Variables	N	%
Gender		
Female	224	84.5
Male	41	15.5
Age		
Under 30 years old	73	27.5
Between 31 and 50 years old	145	54.7
Over 51 years old	47	17.7
Residence		
Rural setting	21	7.9
Urban setting	244	92.1
Residence		
Bachelor or equivalent level	119	44.9
Master, Doctoral or equivalent level	146	55.1
Employee category		
Resident physicians	89	33.6
Specialist physicians	77	29.1
Senior specialist physicians	99	37.4
Marital Status		
Ever married	169	63.8
Never married	96	36.2

Table 2 measured the substance use behaviors during the COVID-19 emergency state and medical examinations requested in this period for certain mental health disorders, as well as relapses for psychiatric conditions during this period. The demographic data delineates substance use behaviors and psychiatric morbidity during the COVID-19 period, offering critical insights. The tabulation reveals a nuanced picture of tobacco use alterations, with 32.8% of participants reporting an increase, contrasting with a 10.9% decrease. Notably, new initiations and cessations of tobacco use were minimal at 3.0% and 2.6%, respectively, while non-smokers constituted a predominant 50.6%. Alcohol consumption patterns depicted a minor segment engaging in heavy use (1.1%), whereas the majority oscillated between light (46.0%) and non-drinking behaviors (42.3%). Psychoactive substance utilization was comparatively infrequent (7.2%). Medical examination requests predominantly aligned with anxious states (11.7%), followed by depressive states (9.4%) and sleep disorders (6.8%), while psychiatric pathology relapses were relatively low at 7.2%.

**Table 2:** Substance use behaviors and psychiatric morbidity during the COVID-19 period

Description	n	%
Change in tobacco use		
Increase in tobacco use	87	32,8
Decrease in tobacco use	29	10,9
Started tobacco use	8	3,0
Quit tobacco use Non-smoker	7 134	2,6 50,6
Alcohol Use Frequency		
Heavy alcohol use (≥ 12 drinks per week)	3	1,1
Moderate alcohol use (6–12 drinks per week)	28	10,6
Light alcohol use (≤ 6 drinks per week)	122	46,0
Non-drinker	112	42,3
Psychoactive substances use		
No	246	92,8
Yes	19	7,2
Request for medical examination		
For anxious states	31	11,7
For sleep disorders	18	6,8
For depressive states	25	9,4
Relapse of psychiatric pathology		
No	246	92,8
Yes	19	7,2

Table 3 provides a comprehensive statistical overview of various psychological measures studied during the COVID-19 period. The Connor-Davidson Resilience Scale (CD-RISC) scores manifest a mean of 66.19, indicative of the resilience levels within the sample. The WHOQOL-Brief domains, ranging from general to environmental areas, elucidate facets of quality of life, with mean scores oscillating between 7.82 in the general area and 28.69 in the environmental area. The COPE inventory delineates coping strategies, categorized into problem-focused, emotion-focused, and dysfunctional coping. Problem-focused coping exhibits an overall mean of 59.72, emphasizing active coping strategies such as planning and the use of instrumental support. Emotion-focused coping, underscored by a mean of 54.54, reveals reliance on emotional support and positive reframing among other strategies. Dysfunctional coping mechanisms, marked by a mean of 39.94, highlight the utilization of various avoidance strategies, including substance use and behavioral disengagement. Each measure, complemented by the presentation of mean, median, and interquartile range values, provides a nuanced understanding of the participants’ psychological adaptation during the COVID-19 period.

**Table 3:** Summary of Physicians' Resilience, Quality of Life, and Coping Strategies in Response to COVID-19 Stressors

Studied measures	Mean ± SD	Median (Min–Max)	Interquartile range
CD-RISC	66.19 ± 14.42	67 (22–99)	18.5
WHOQOL-BREF			
Physical area (7 items)	64.00 ± 14.21	64.28 (21.43–96.43)	5.5
Mental area (6 items)	61.71 ± 16.17	62.50 (12.50–100)	6
Social Relations area (3 items)	61.22 ± 20.25	66.66 (0–100)	3
Environment area (8 items)	64.65 ± 12.71	65.62 (21.88–100)	5.5
COPE inventory			
Problem-focused coping	59.72 ± 7.47	59 (38–79)	9.5
Active coping	12.64 ± 1.98	12 (7–16)	3
Planning	13.12 ± 2.14	13 (8–16)	3
Use of instrumental support	11.99 ± 2.36	12 (5–16)	3
Restraint	10.80 ± 2.07	11 (5–16)	2
Suppression of competing activities	11.18 ± 2.18	11 (5–16)	2
Emotion-focused coping	54.54 ± 8.64	55 (35–79)	12.5
Use of emotional support	11.26 ± 3.09	11 (4–11)	4
Positive reframing	12.48 ± 2.13	12 (6–16)	3
Acceptance	11.63 ± 2.33	12 (5–16)	3
Religious	9.02 ± 4.17	9 (4–16)	8
Humor	10.15 ± 3.58	10 (4–16)	5
Dysfunctional coping	39.94 ± 7.86	39 (23–79)	9
Denial	6.45 ± 2.13	6 (4–16)	3
Substance use	5.86 ± 3.03	4 (4–16)	4
Behavioural disengagement	7.43 ± 2.14	8 (4–16)	2
Mental disengagement	10.06 ± 2.35	10 (4–16)	3
Venting	10.14 ± 2.55	10 (4–16)	4

The Connor-Davidson Resilience Scale (CD-RISC) has a maximum score of 100. The domains of the WHOQOL-BREF have a maximum score of 100. Each subscale of the COPE Inventory has a maximum sxore of 16

The data provided in Table 4 aims to elucidate the relationship between various variables and levels of resilience, as measured by the Connor-Davidson Resilience Scale (CD-RISC 25). In the univariate analysis, problem-focused coping was positively associated with resilience levels (B = 0.855, 95% CI [0.645, 1.065], p < 0.001***). This association remained significant in the multivariate analysis, though attenuated (B = 0.370, 95% CI [0.147, 0.594], p = 0.001**). A significant positive relationship was observed between emotion-focused coping and resilience levels, both in univariate (B = 0.754, 95% CI [0.573, 0.935], p < 0.001***) and multivariate analyses (B = 0.689, 95% CI [0.492, 0.886], p < 0.001***). Dysfunctional coping exhibited a negative association with resilience levels in the univariate (B = -0.481, 95% CI [-0.696, -0.266], p < 0.001***) and multivariate analyses (B = − 0.567, 95% CI [− 0.748, − 0.385], p < 0.001***). Being male, as opposed to female (reference), was significantly associated with higher resilience levels in univariate analysis (B = 6.462, 95% CI [1.695, 11.229], p = 0.008**), with this relationship maintaining significance in multivariate analysis (B = 4.444, 95% CI [0.613, 8.276], p = 0.023*). Different age groups exhibited varied levels of resilience. Individuals under 30 showed lower resilience levels in univariate analysis (B = − 4.865, 95% CI [− 8.731, − 0.999], p = 0.014*), but this association was not significant in multivariate analysis. Having a master’s or doctoral degree, compared to a bachelor’s (reference), was positively associated with resilience levels in univariate analysis (B = 6.030, 95% CI [2.595, 9.466], p = 0.001**), but lost significance in the multivariate analysis. No significant association was found between urban or rural residential location and resilience levels in either univariate or multivariate analysis. Unmarried individuals showed lower resilience levels in univariate analysis (B = − 4.554, 95% CI [− 8.147, − 0.962], p = 0.013*), but this was not significant in multivariate analysis. Variations in resilience were observed based on professional status. Resident physicians showed lower resilience in univariate analysis (B = − 5.641, 95% CI [− 9.277, − 2.006], p = 0.002**), but this relationship was not significant in multivariate analysis. Senior specialists showed higher resilience in univariate analysis (B = 6.637, 95% CI [3.116, 10.157], p < 0.001***), with the association trending towards significance in the multivariate analysis (B = 3.549, 95% CI [− 0.196, 7.295], p = 0.063).

**Table 4:** Univariate and multivariate linear regression for levels of resilience measured by CD-RISC 25

Variables	Univariate analysis		Multivariate analysis	
	B (95% CI)	p-value	B (95% CI)	p-value
Problem-focused Coping	0.855 (0.645, 1.065)	< 0.001***	0.370 (0.147, 0.594)	0.001**
Emotion-focused Coping	0.754 (0.573, 0.935)	< 0.001***	0.689 (0.492, 0.886)	< 0.001***
Dysfunctional Coping	− 0.481 (− 0.696, − 0.266)	< 0.001***	− 0.567 (− 0.748, − 0.385)	< 0.001***
Males (ref. Females)	6.462 (1.695, 11.229)	0.008**	4.444 (0.613, 8.276)	0.023*
Under 30 years old (Ref. More than 30)	− 4.865 (− 8.731, − 0.999)	0.014*	2.214 (− 3.449, 7.876)	0.442
Over 51 years old (Ref. less than 51)	4.520 (− 0.021, 9.062)	0.051	1.106 (− 2.996, 5.207	0.596
Masters/Doctoral Degree (Ref. Bachelors')	6.030 (2.595, 9.466)	0.001**	1.006 (− 2.296, 4.307	0.600
Urban (Ref. Rural)	0.730 (− 5.737, 7.198)	0.824	− 1.417 (− 6.527, 3.693)	0.585
Unmarried (Ref. Married)	− 4.554 (− 8.147, − 0.962	0.013*	− 1.674 (− 4.991, 1.643)	0.321
Resident physicians (Ref. Specialists and Seniors)	− 5.641 (− 9.277, − 2.006)	0.002**	− 2.398 (− 8.042, 3.246)	0.404
Senior Specialists (Ref. Residents and Specialists)	6.637 (3.116, 10.157)	< 0.001***	3.549 (− 0.196, 7.295)	0.063

In this table, "B" represents the unstandardized coefficients, and CI stands for Confidence Interval. Significance level marked as "*" for p < 0.05, "**" for p < 0.01, and "***" for p < 0.001

The univariate and multivariate linear regression analyses were conducted to examine the relationships between several variables and the four domains of QOL measured by the WHO QOL-BREF. The variables included CD-RISC scores, problem-focused coping, emotion-focused coping, dysfunctional coping, and several demographic factors. The results of these analyses are presented in the Table 5.

**Table 5:** Univariate and Multivariate linear regression for variables predicting the four Domains of QOL

Variables	WHO QOL-BREF	Univariate analysis		Multivariate analysis	
		B (95% CI)	p-value	B (95% CI)	p-value
CD-RISC Scores	Physical	0.43 (0.32, 0.54)	< 0.001***	0.32 (0.18, 0.45)	< 0.001***
	Psychological	0.68 (0.58, 0.79)	< 0.001***	0.49 (0.35, 0.63)	< 0.001***
	Relationship	0.63 (0.48, 0.78)	< 0.001***	0.49 (0.29, 0.68)	< 0.001***
	Environment	0.43 (0.34, 0.52)	< 0.001***	0.37 (0.25, 0.50)	< 0.001***
Problem-focused Coping	Physical	0.31 (0.09, 0.54)	0.007**	− 0.14 (− 0.39, 0.11)	0.268
	Psychological	0.73 (0.48, 0.98)	< 0.001***	0.05 (− 0.21, 0.30)	0.710
	Relationship	0.72 (0.40. 1,04)	< 0.001***	0.10 (− 0.26, 0.45)	0.585
	Environment	0.46 (0.26, 0.66)	< 0.001***	0.08 (− 0.15, 0.31)	0.496
Emotion-focused Coping	Physical	0.41 (0.21, 0.60)	< 0.001***	0.28 (0.05, 0.52)	0.019*
	Psychological	0.71 (0.50. 0.92)	< 0.001***	0.37 (0.13, 0.61)	0.003**
	Relationship	0.80 (0.53, 1,06)	< 0.001***	0.38 (0.05, 0.72)	0.024*
	Environment	0.41 (0.24, 0.58)	< 0.001***	0.08 (-0.13, 0.29)	0.460
Dysfunctional Coping	Physical	− 0.52 (− 0.73, − 0.31)	< 0.001***	− 0.47 (− 0.68, − 0.25)	< 0.001***
	Psychological	− 0.53 (− 0.77, − 0.29)	< 0.001***	− 0.36 (− 0.58, − 0.14)	0.001**
	Relationship	− 0.41 (− 0.72, − 0.10)	0.010**	− 0.30 (− 0.60. 0.00)	0.053
	Environment	− 0.18 (− 0.37, 0.02)	0.076	0.00 (− 0.19, 0.19)	0.998
Males (ref. Females)	Physical	4.66 (− 0.07, 9.39)	0.053	2.39 (− 1.88, 6.66)	0.271
	Psychological	7.30 (1.96, 12.65)	0.008**	3.33 (− 1.01, 7.67)	0.132
	Relationship	3.31 (− 3,47, 10.09)	0.337	− 0.63 (− 6.67, 5.40)	0.836
	Environment	3.58 (− 0.66, 7.82)	0.098	0.44 (− 3.42, 4.30)	0.823
Under 30 years old (Ref. More than 30)	Physical	1.40 (− 2.45, 5.25)	0.474	1.49 (− 4.76, 7.74)	0.639
	Psychological	− 5.14 (− 9.48, − 0.80)	0.021*	− 1.78 (− 8.13, 4.58)	0.582
	Relationship	3.10 (− 2.39, 8.58)	0.267	− 0.79 (− 9.63, 8.05)	0.861
	Environment	− 2.21 (− 5.65, 1.23)	0.207	− 0.69 (− 6.35, 4.97)	0.811
Over 51 years old (Ref. less than 51)	Physical	− 1.78 (− 6.28, 2.72)	0.437	− 3.09 (− 7.62, 1.43)	0.179
	Psychological	2.90 (− 2.22, 8.01)	0.266	− 1.22 (− 5.82, 3.38)	0.602
	Relationship	− 3.30 (− 9.72, 3,11)	0.312	− 4.61 (− 11.01, 1.79)	0.157
	Environment	0.85 (− 3.18, 4.89)	0.678	− 2.07 (− 6.17, 2.02)	0.320
Masters/Doctoral Degree (Ref. Bachelors')	Physical	3.14 (− 0.31, 6.58)	0.074	2.89 (− 0.76, 6.53)	0.12
	Psychological	6.20 (2.33, 10.07)	0.002**	1.22 (− 2.49, 4.92)	0.518
	Relationship	3.98 (− 0.93, 8.89)	0.112	4.81 (− 0.34, 9.96)	0.067
	Environment	4,44 (1.39, 7.49)	0.004**	2.12 (− 1.18, 5.42)	0.206
Urban (Ref. Rural)	Physical	− 1.05 (− 7.42, 5.33)	0.747	− 2.22 (− 7.85, 3.42)	0.439
	Psychological	0.87 (− 6.38, 8.12)	0.813	− 0.50 (− 6.23, 5.23)	0.863
	Relationship	1.42 (− 7.67, 10.50)	0.759	− 1.05 (− 9.02, 6.92)	0.796
	Environment	− 0.89 (− 6.59, 4.82)	0.759	− 1.58 (− 6.69, 3.52)	0.542
Unmarried (Ref. Married)	Physical	− 0.08 (− 3.66, 3.50)	0.964	0.84 (− 2.83, 4.50)	0.653
	Psychological	− 3.94 (− 7.99, 0.11)	0.056	− 0.02 (− 3.75, 3.70)	0.991
	Relationship	− 2.36 (− 7.46, 2.74)	0.363	− 3.53 (− 8,71, 1.66)	0.182
	Environment	− 2.82 (− 6,01, 0.37)	0.082	− 1.35 (− 4.67, 1.96)	0.422
Resident physicians (Ref. Specialists and Seniors)	Physical	0.97 (− 2.67, 4.61)	0.601	3.61 (− 2.62, 9.84)	0.255
	Psychological	− 5,09 (− 9.19, − 0.99)	0.015**	1.35 (− 4.99, 7.68)	0.676
	Relationship	4.38 (− 0.79, 9.56)	0.096	12.17 (3.35, 20.98)	0.007**
	Environment	− 2.09 (− 5.34, 1.17)	0.208	3.37 (− 2.27, 9.01)	0.241
Senior Specialists (Ref. Residents and Specialists)	Physical	1.89 (− 1.66, 5.44)	0.295	1.63 (− 2.52, 5.79)	0.439
	Psychological	6.36 (2.39, 10.34)	0.002**	2.23 (− 2.00, 6.46)	0.300
	Relationship	0.62 (− 4.45, 5.70)	0.809	1.59 (− 4.29, 7.47)	0.596
	Environment	4.06 (0.92, 7.21)	0.012*	2.53 (− 1.23, 6.30)	0.186

In this table, "B" represents the unstandardized coefficients, and CI stands for Confidence Interval. Significance level marked as "*" for p < 0.05, "**" for p < 0.01, and "***" for p < 0.001

In the univariate analysis, CD-RISC scores were positively associated with all four domains of QOL (Physical: B = 0.43, 95% CI [0.32, 0.54], p < 0.001***; Psychological: B = 0.68, 95% CI [0.58, 0.79], p < 0.001***; Relationship: B = 0.63, 95% CI [0.48, 0.78], p < 0.001***; Environment: B = 0.43, 95% CI [0.34, 0.52], p < 0.001***). Problem-focused coping was positively associated with the physical domain (B = 0.31, 95% CI [0.09, 0.54], p = 0.007**), psychological domain (B = 0.73, 95% CI [0.48, 0.98], p < 0.001***), relationship domain (B = 0.72, 95% CI [0.40, 1.04], p < 0.001***), and environment domain (B = 0.46, 95% CI [0.26, 0.66], p < 0.001***) in the univariate analysis. In the multivariate analysis, however, problem-focused coping was not significantly associated with any of the domains of QOL.

Emotion-focused coping was significantly and positively associated with all QOL domains in the univariate analysis (Physical: B = 0.41, 95% CI [0.21, 0.60], p < 0.001***; Psychological: B = 0.71, 95% CI [0.50, 0.92], p < 0.001***; Relationship: B = 0.80, 95% CI [0.53, 1.06], p < 0.001***; Environment: B = 0.41, 95% CI [0.24, 0.58], p < 0.001***). In the multivariate analysis, emotion-focused coping remained significantly associated with the physical domain (B = 0.28, 95% CI [0.05, 0.52], p = 0.019*), psychological domain (B = 0.37, 95% CI [0.13, 0.61], p = 0.003**), and relationship domain (B = 0.38, 95% CI [0.05, 0.72], p = 0.024*), but not with the environment domain.

Dysfunctional coping was significantly associated with the physical domain (B =− 0.52, 95% CI [-0.73, -0.31], p < 0.001***), psychological (B =− 0.53, 95% CI [-0.77, -0.29], p < 0.001***), and relationship domains (B =− 0.41, 95% CI [-0.60, -0.21], p < 0.001***) in the univariate analysis. In the multivariate analysis, dysfunctional coping remained significantly and negatively associated with the psychological domain (B =− 0.31, 95% CI [-0.58, -0.03], p = 0.031*), but not with the physical or relationship domains.

In the univariate analysis, gender was significantly associated with the psychological domain of WHO QOL-BREF, with males having higher scores than females (B = 7.30, 95% CI [1.96, 12.65], p = 0.008**). Participants under 30 had significantly lower scores in the psychological domain than those over 30 (B =− 5.14, 95% CI [-9.48, -0.80], p = 0.021*). Individuals with a master's or doctoral degree had significantly higher scores in the psychological (B = 6.20, 95% CI [2.33, 10.07], p = 0.002**) and environment (B = 4.44, 95% CI [1.39, 7.49], p = 0.004**) domains compared to those with a bachelor's degree. Resident physicians had significantly lower scores in the psychological domain than specialists and senior specialists (B =− 5.09, 95% CI [-9.19, -0.99], p = 0.015). The multivariate analysis found no significant association between gender and the physical, relationship, and environment domains. The psychological domain was not significantly associated with age or education level in the multivariate analysis. Resident physicians showed a significant positive association with the relationship domain compared to specialists and senior specialists (B = 12.17, 95% CI [3.35, 20.98], p = 0.007**). No other significant associations were found in the multivariate analysis.

## Discussion

The objective of our study was to thoroughly analyze how various factors contribute to the resilience and quality of life (QOL) of Romanian physicians during the COVID-19 emergency in 2021. Utilizing the COPE inventory to assess coping strategies, we examined the association between coping strategies and QOL domains and resilience. Our cross-sectional approach also explored the interplay between demographic factors, coping strategies, and their overall association with resilience and the WHO's four QOL domains. Moreover, we aimed to investigate the role of resilience in influencing these QOL areas and to document substance use and psychiatric morbidity during this period.

The COVID-19 pandemic has had a significant impact on physicians' mental health, resulting in heightened levels of stress, anxiety, depression, and fatigue. This is primarily due to the increased work demands, limited opportunities for recovery, and high-intensity, time-pressured working patterns associated with managing the pandemic [41]. High rates of depression, distress, and suicidal thoughts in the HCWs during the lockdown were alarming [42]. It is crucial for healthcare systems to provide short and long-term psychological support for physicians caring for patients during infectious disease outbreaks [43].

Physicians and other healthcare workers have an important role during times of public health crises, such as the COVID‐19 pandemic. We investigated the main coping and resilience mechanisms and the role of positive and negative stress-reducing activities on mental and physical well-being in Romanian HCWs. Our findings regarding demographic characteristics (mean age) and resilience score are in line with other published reports on mental health outcomes among healthcare workers exposed to the COVID‐19 pandemic [44, 45]. Our cohort is represented by a majority of female physicians, which in turn represent a small part of all female Romanian physicians [46]. Although 84.5% of physicians from our sample are females and 27.5% are under 30 years old, this is in line with the European healthcare personnel statistics, which show that in 2021, more than 70% of the total number of physicians in Romania were women, with 34.4% of Romanian physicians being under 35 years old and only 20.4% of physicians were aged 55 years and over in 2021 [47]. Problem-focused and emotion-focused coping strategies were positively associated with higher resilience and with better quality of life across several domains, whereas dysfunctional coping had a negative association. The persistent negative association of dysfunctional coping with the physical and psychological domain of QOL and resilience in the multivariate analysis was notable, as it implies a consistent detrimental effect regardless of other factors. The interrelationship between coping mechanisms, resilience, and quality of life suggests that interventions aimed at improving coping skills could enhance overall life satisfaction and well-being during crises. A review conducted in 2021 has revealed that effective coping mechanisms are essential for managing emergencies and related occupational stress. Negative coping strategies such as escape and avoidance mechanisms or overcommitment have been linked to poor mental health outcomes. On the other hand, maintaining a positive attitude towards the problem, having a strong social network, receiving peer support, working in a team, being self-reliant, negotiating problems efficiently, and practicing self-care can all help to reduce stress levels and improve resilience [48]. The data indicated a nuanced shift in substance use, with an increase in tobacco use for nearly one-third of participants, which might reflect stress-related behaviors during COVID-19. In 2020, Vanderbruggen et al. [49] found that overall alcohol consumption and cigarette smoking increased during the lockdown, with reasons including boredom and lack of social contacts In 2021, Mounir I et al. [50] reported that hospital workers who experienced increased sadness and distress during the lockdown were more likely to use tobacco. Giovenco et al. [51] highlighted that changes in tobacco use patterns were driven by individual-level factors such as anxiety and irregular routines, as well as limited access to alternative products like electronic nicotine delivery systems. These findings collectively suggest a nuanced shift in substance use, with an increase in tobacco use potentially linked to stress-related behaviors during the COVID-19 pandemic.

Effective coping strategies, whether problem-focused or emotion-focused, might serve as mediators in the relationship between stressors experienced during the COVID-19 pandemic and mental health outcomes. Gender differences in resilience might be attributed to social, behavioral, or biological factors that contribute to how individuals respond to stress.

Additionally, in our previous paper about coping mechanisms and resilience in psychiatric trainees during the pandemic, we also found that good psychological mechanisms and adaptiveness decrease vulnerability to impairment [52]. Crișan et al., found similar results regarding the negative relation between dysfunctional coping and physical health among Romanian citizens during the current war in Ukraine [53]. From our knowledge, there is no research data regarding the correlation between coping strategies evaluated using COPE inventory and quality of life evaluated using WHOQOL-BREF among Romanian HCWs during the COVID-19 pandemic. Therefore, further research is needed in this field.

This study provides insights into the impact of the COVID-19 pandemic on dysfunctional coping mechanisms, which in turn have an impact on certain life quality domains. Nurturing improved coping abilities and resilience may contribute to beneficial healthcare workforce mental health. These findings advocate for the implementation of organizational interventions tailored to cultivate resilience and improved coping behaviors, utilizing counseling, fostering social connections, and specific resilience training. Such interventions could include the establishment of trauma risk management programs to empower workers to support their peers, thus nurturing a thriving work environment. Additionally, incorporating practices such as self-care routines, group discussions, mindfulness sessions, and targeted training can be instrumental in fostering resilience, ultimately contributing to a more robust and adaptive workforce [48]. The findings could inform public health strategies and interventions aimed at supporting mental health during emergency situations.

The study, while offering valuable insights into substance use, mental health disorders, and coping mechanisms during the COVID-19 emergency state, is not without limitations. Firstly, the cross-sectional nature of the study precludes the establishment of causality; longitudinal studies would be needed to discern the temporal relationships between the pandemic, substance use behaviors, and the development of psychiatric conditions. Secondly, the reliance on self-reported data could introduce recall bias or social desirability bias, potentially skewing the reported prevalence of substance use and mental health symptoms. Thirdly, the sample may not be representative of the broader population, as it might over-represent individuals with internet access or those more inclined to participate in research studies, limiting the generalizability of the findings. Furthermore, while the study provides associations between coping mechanisms and resilience, it does not account for all potential confounding variables such as previous mental health history, socioeconomic status, or access to healthcare services, which could significantly influence these outcomes. Finally, the study's focus on certain demographic factors (like education level, gender, marital status) might overlook other critical variables (like ethnicity, specific occupational stressors, or pre-existing health conditions) that might have offered a more nuanced understanding of the population’s mental health and coping behaviors during the pandemic. These limitations suggest a need for cautious interpretation of the results and an impetus for future research that addresses these gaps.

## Conclusions

The study elucidates a multifaceted picture of how the COVID-19 pandemic impacted substance use, mental health, and coping mechanisms among a sample of participants. The study suggests that promoting adaptive coping strategies, providing robust support for mental health, and understanding the demographic variables that influence resilience are critical in mitigating the negative effects of a pandemic on the mental health and quality of life of physicians. These findings offer guidance for healthcare providers, policymakers, and public health practitioners to develop tailored interventions aimed at supporting populations during times of crisis. Future research should focus on developing better measures of resilience that would capture constructs that are unique to pandemics.

## Data Availability

The datasets used and analyzed during the current study are available from the University of Medicine and Pharmacy "Iuliu Hațieganu". Data are available from the authors upon reasonable request and with the permission of the University of Medicine and Pharmacy "Iuliu Hațieganu".

## Abbreviations

COVID-19: Coronavirus infectious disease 2019
COPE: Coping Orientation to Problems Experienced
CD-RISC 25: Connor-Davidson Resilience Scale 25
WHOQOL-BREF: The World Health Organization Quality of Life Brief Version
QOL: Quality of life
WHO: The World Health Organization
HCWs: Healthcare workers
SARS-CoV-2: Severe acute respiratory syndrome coronavirus 2
